# Artificial neural networks for linkage analysis of quantitative gene expression phenotypes and evaluation of gene × gene interactions

**DOI:** 10.1186/1753-6561-1-s1-s47

**Published:** 2007-12-18

**Authors:** Ying Liu, Weimin Duan, Justin Paschall, Nancy L Saccone

**Affiliations:** 1 Department of Genetics, Washington University School of Medicine, 4566 Scott Avenue, Box 8232, St. Louis, Missouri 63110, USA

## Abstract

**Background:**

Using single-nucleotide polymorphism (SNP) genotypes and selected gene expression phenotypes from 14 CEPH (Centre d'Etude du Polymorphisme Humain) pedigrees provided for Genetic Analysis Workshop 15 (GAW15), we analyzed quantitative traits with artificial neural networks (ANNs). Our goals were to identify individual linkage signals and examine gene × gene interactions. First, we used classical multipoint methods to identify phenotypes having nominal linkage evidence at two or more loci. ANNs were then applied to sib-pair identity-by-descent (IBD) allele sharing across the genome as input variables and squared trait sums and differences for the sib pairs as output variables. The weights of the trained networks were analyzed to assess the linkage evidence at each locus as well as potential interactions between them.

**Results:**

Loci identified by classical linkage analysis could also be identified by our ANN analysis. However some ANN results were noisy, and our attempts to use cross-validated training to avoid overtraining and thereby improve results were only partially successful. Potential interactions between loci with high-ranked weight measures were also evaluated, with the resulting patterns suggesting existence of both synergistic and antagonistic effects between loci.

**Conclusion:**

Our results suggest that ANNs can serve as a useful method to analyze quantitative traits and are a potential tool for detecting gene × gene interactions. However, for the approach implemented here, optimizing the ANNs and obtaining stable results remains challenging.

## Background

Complex traits are often hypothesized to be influenced by multiple interacting loci. Detecting gene × gene interactions remains a challenge. Artificial neural networks (ANNs), however, are suited for pattern recognition involving combinations of loci.

ANNs were first applied for human linkage analysis in Genetic Analysis Workshop 10 (GAW) [1]. We have previously used ANNs to identify loci linked to discrete disease traits simulated for GAW11 [2]. Others have applied ANNs for linkage [3,4] or association analysis [5]. For GAW15, we extend our ANN method to quantitative traits, and aim to identify not only linked loci but also potential interactions between loci.

## Methods

The GAW15 Problem 1 data set, from Morley et al. [6], includes single-nucleotide polymorphism (SNP) genotypes at 2882 loci across the genome and 3554 gene expression phenotypes in lymphoblastoid cells, for 14 three-generation CEPH (Centre d'Etude du Polymorphisme Humain) Utah families.

To select phenotypes for ANN analysis, we carried out Haseman-Elston (H-E) regression [7] using the Statistical Analysis for Genetic Epidemiology package (S.A.G.E., Release 5.2: ) and variance-components (VC) linkage analysis using Merlin [8]. The former analysis is the same as that used by Morley et al. [6].

We used two different genetic maps. We compiled Map 1 from the SNP Consortium map data [9]. Map 2 was provided by Ellen Wijsman's group [10]. Comparing the two maps, we found that Map 2 included a greater number of the GAW15 markers. Early ANN analyses used Map 1 and were then updated using Map 2; we report the latter results, and describe some contrasts with Map 1 results in the Discussion. Identity-by-descent (IBD) sharing was estimated across the genome for 378 sib pairs using Merlin. IBD sharing at 10-cM gridpoints was presented as input data to the ANNs, coded as a continuous variable between 0 and 1. Phenotype data was used as the output values at two output nodes: the first for the squared trait difference, and the second for the mean-corrected trait sum. This coding thus contains the key phenotypic information also used by the "new" H-E method [11].

We used the Stuttgart Neural Network Simulator (SNNS), Version 4.2 [12]. ANN training used standard back-propagation with weight decay. Initial runs used all sib pairs for both training and validation, similar to the method of Lucek and colleagues [1,3]; after a fixed number of cycles, the trained net having lowest sum-of-squares-error (SSE) between network outputs and data-specified target outputs underwent further analysis of its weights. Subsequently, we used five-fold cross-validated training to avoid overtraining and improve the generalization ability of the ANN models; we also hoped that cross-validated models would lead to more stable linkage results. An important feature of our previous ANN work was our use of cross-validation to select models before the ANN weights were analyzed for linkage evidence [2]. We hypothesized that cross-validation would also help address concerns about unstable ANN linkage results such as reported in [13].

We used a network architecture with two hidden layers. We found that two hidden layers performed better than a single layer, attaining lower error and more appropriate output values. Note that the phenotype coding allows the squared trait sums and differences to range freely. Thus, the activation function at the output nodes was the identity function rather than the usual logistic sigmoid function, since the latter would restrict outputs to range between 0 and 1. We believe the second hidden layer is therefore performing some rescaling needed to obtain appropriate output values. Thus, the primary ANNs contained 362 input nodes, two hidden layers of 50 nodes each, and two output nodes.

To identify input loci that are important in determining phenotypic status, we used an algorithm similar to those in our previous work [2]. Consider a trained network with *I *input nodes, two hidden layers with *H *nodes each, and *O *output nodes, such that each node in a given layer is connected to every node in the next layer. Suppose the *i*^th ^input *x*_*i *_is connected to the *j*^th ^hidden node via a weight *u*_*ij*_, the *j*^th ^hidden node is in turn connected to the *k*^th ^hidden node in the next layer via a weight *v*_*jk*_, and this *k*^th ^hidden node is connected to the *l*^th ^output node via *w*_*kl*_. Then we calculate an "importance measure" for the *i*^th ^input: $\sum_{l=1}^{O}\sum_{k=1}^{H}\sum_{j=1}^{H}|u_{ij}v_{jk}w_{kl}|$. To combine results across the five separate ANNs generated by cross-validated analyses, we standardized the importance measure by subtracting the mean and dividing by the standard deviation, then averaged across the five ANNs to rank the signals at each input.

For our novel analysis of the interaction between a given pair of loci, we considered the weights for each pair of connections leading from the two loci to the same node in the next, hidden layer. We calculated the Pearson correlation for the resulting data set of 50 pairs of weights. A strong positive correlation is interpreted as cumulative or synergistic action of the loci, and a strong negative correlation may be interpreted as either antagonistic or complementary action. The rationale for this correlation analysis relies on the following details of an ANN's operation. For the generic network described above, a linear combination of the *I *inputs *x*_*i *_forms the argument for the activation function *f*_*j *_of the *j*^th ^node of the next layer, with weights *u*_*ij *_as the coefficients, as follows: $f_{j}\left(a_{j}+{\sum_{i=1}^{I}u_{ij}x}_{i}\right)$, where *a*_*j *_is an intercept term. We hypothesized that if two inputs have positively correlated weights leading to the next layer, similar input values will have cumulative effect on the arguments of the activation functions; for two inputs with negatively correlated weights, similar input values will have antagonistic effect. A simplified example, in which the weights from one input node are a fixed multiple of those from a second, unlinked input node and thus are perfectly correlated with those latter weights, demonstrates this most clearly. Letting *c *be the scaling factor for the weights and indexing these two inputs with 1 and 2, then *u*_2*j *_= *cu*_1*j *_for all *j*, and the *j*^th ^activation function value is: $f_{j}\left(a_{j}+u_{1j}(x_{1}+cx_{2})+\sum_{i=3}^{I}u_{ij}x_{i}\right)$. In the extreme case in which |*c*| = 1, we see that when *c *is positive, similar input values will have cumulative effects across all activation functions in the hidden layer, and when *c *is negative, subtraction will cancel out the effects of two similar input values.

To further evaluate potential interactions detected by our correlation analysis, we used both VC (SOLAR with the "-epistasis" option [14]) and a simple categorical "bin" analysis. For the latter, we subdivided the sibpairs into a three-by-three table of low (0 ≤ IBD < 1/3) medium (1/3 ≤ IBD < 2/3) and high (2/3 ≤ IBD ≤ 1) IBD categories at each locus in the pair. In each cell the proportion of "trait dissimilar" sib pairs was calculated, where pairs were labeled dissimilar if their squared trait difference was greater than the overall average. This descriptive analysis helped us assess our hypotheses about the effects of positively versus negatively correlated weights.

## Results

We selected two gene expression phenotypes for ANN analysis: probe sets 203313_s_at (gene *TGIF*, TGFB-induced factor) and 210910_s_at (gene *ZP3*, zona pellucida glycoprotein 3). These traits gave at least two distinct H-E signals with *p*-values less than 0.0001, and had consistent VC results (Table 1). Probe set 203313_s_at was also reported to have multiple linkage peaks by Morley et al. [6].

**Table 1:** Selected gene expression phenotypes

Probe set	Gene	H-E *p*-value	H-E Location	VC *p*-value	VC location
203313_s_at	*TGIF*	8.8 × 10^-12^	Chr 1: 28 cM	4.00 × 10^-5^	Chr 1: 27 cM
		4.8 × 10^-6^	Chr 15: 22 cM	5.00 × 10^-3^	Chr 15: 25 cM
		2.00 × 10^-5^	Chr 9: 127 cM	-	-
		2.21 × 10^-5^	Chr 9: 14 cM	-	-
					
210910_s_at	*ZP3*	3.75E × 10^-16^	Chr 7: 83 cM	0.0	Chr 7: 83 cM
		1.24 × 10^-5^	Chr 15: 41 cM	3.00 × 10^-3^	Chr 15: 41 cM
		4.00 × 10^-4^	Chr 12: 103 cM	-	-
		1.80 × 10^-3^	Chr 14: 103 cM	4.00 × 10^-3^	Chr 14: 103 cM

**Figure 1:**
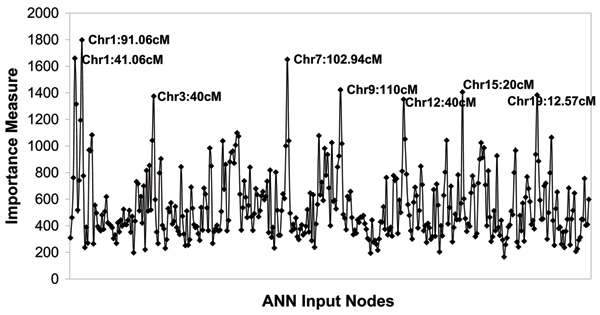
ANN analysis of 203313_s_at (*TGIF*), using Map 2 and all sib pairs.

ANN analysis of *TGIF *with Map 2 detected linkage on chromosomes 1, 9, and 15, as expected from the H-E and VC results; however, results were noisy with multiple other putative peaks (Fig. 1). An interesting pattern of interaction was suggested by the correlation analysis, which revealed strong positive correlation between the chromosomes 1 and 15 signals (*r *= 0.961), and strong negative correlation between the chromosomes 1 and 9 signals (*r *= -0.896) (Table 2). These pairings were ranked second and nineteenth, respectively, across all pairings of the chromosome 1 locus with each 10-cM gridpoint across the map.

**Table 2:** Correlation analysis of two-locus interactions using the ANN weights

Phenotype	Input 1^*a*^	Input 2^*a*^	Pearson *r*	Rank of |r|
203313_s_at (*TGIF*)	Chr 1: 40 cM	Chr 15: 20 cM	0.961	2
	Chr 1: 40 cM	Chr 9: 110 cM	-0.896	19
				
210910_s_at (*ZP3*)	Chr 7: 90 cM	Chr 7: 83 cM	0.957	1
	Chr 7: 90 cM	Chr 15: 40 cM	0.703	3

^*a *^Loci with high ANN importance measure, and supported by H-E and VC analysis.

**Figure 2:**
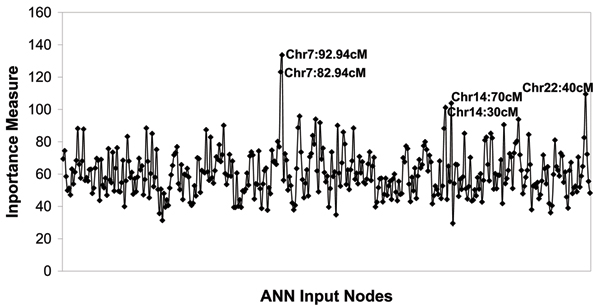
ANN analysis of 210910_s_at (*ZP3*), using Map 2 and all sib pairs.

ANN results for 210910_s_at (*ZP3*) using Map 2 are given in Figure 2. The highest peak identified by the ANN is consistent with the strongest linkage peak from both H-E and VC analyses, and the secondary peak on chromosome 14 matches one of the additional notable linkage peaks. Correlation results are given in Table 2. Interestingly, although chromosome 15 (the second best signal from traditional analysis) was not detected by the ANN method (Fig. 2), the interaction analysis highlighted a strong correlation with the chromosome 7 locus.

We subjected the most interesting locus pairings from our weight correlation analysis of *TGIF *to epistasis analysis in a VC framework, and also to a simple categorical "bin" analysis. The VC analysis did estimate a non-zero coefficient for the epistasis term, but this did not significantly improve the log-likelihood fit compared with sequential single locus scanning. However, our bin analysis revealed interesting patterns. We had observed a strong positive correlation for chromosome 1, 40 cM and chromosome 15, 20 cM (Table 2); correspondingly, the highest ratio of dissimilar pairs (66%) occurred in the bin corresponding to low sharing at *both *loci, consistent with our hypothesis of cumulative effect. In contrast, for the pairing that gave a strong negative correlation (chromosome 1, 40 cM and chromosome 9, 110 cM), the highest ratio of dissimilar pairs (56%) was in the bin corresponding to low sharing at the first locus and high sharing at the second. Thus, while from H-E we expect low trait similarity among low IBD sib pairs at each locus, considering the IBD status jointly reveals an interaction. The negative correlation was observed in the presence of an underlying pattern in which high sharing at one locus together with low sharing at the other led to reduced trait similarity.

For the computationally intensive cross-validated analyses, we targeted only TGIF. We completed three five-fold cross-validated runs and observed whether the H-E/VC verified loci were supported. The first run supported the loci at chromosome 1, 40 cM (rank = 7); chromosome 9, 110 cM (rank = 4); and chromosome 15, 20 cM (rank = 8). The second supported only the chromosome 9 locus (rank = 4), and the third supported chromosome 9 (rank = 6) and chromosome 15 (rank = 5), with evidence on chromosome 1, 90 cM (rank = 1). Other high-ranking loci were not always in common across the three runs. Thus these results were not as consistent as expected; however, they did provide some support for the key loci.

We performed secondary analyses of rescaled data (inputs and outputs), but results did not notably improve. Based on our results, we did not further develop those approaches.

## Discussion and conclusion

Currently, genome-wide linkage scans are typically carried out one chromosome at a time. Approaches that can analyze all markers simultaneously and detect patterns of locus interactions are a desired alternative. We applied ANNs to map QTLs and calculated correlations between the ANN weights to evaluate potential interactions.

Training and testing ANNs on all sib pairs (rather than using cross-validation) did detect linkage evidence at loci highlighted by traditional methods. We favor the use of cross-validation when possible [2]. We applied five-fold cross-validation to select models; however, this approach did not appear to ultimately improve stability of the linkage results: the first cross-validated analysis supported the loci detected traditional analysis; subsequent cross-validated results were not as consistent with each other as we had hoped. Given our previous success in using cross-validated ANNs for analysis of discrete traits [2], we speculate that quantitative traits may present a greater challenge for ANN-based linkage analysis. It is also possible that an alternative coding of the quantitative phenotype data would have been more suitable than what we used here. Alternative ANN configurations were considered and trained on the data. Although using 50 nodes for the hidden layers was somewhat arbitrary, alternative configurations did not seem to improve performance. Nevertheless, choice of architecture remains an issue for ANN applications.

Our initial ANN analysis run used the 203313_s_at (*TGIF*) gene expression phenotype, Map 1, and a randomly selected training subset of four-fifths of the sib pairs (data not shown). This preliminary analysis was promising because it detected the expected linkage peaks on chromosomes 1, 9, and 15, and only one additional unconfirmed peak. Furthermore, the correlation analysis was even more striking (*r *= 0.985 and -0.917), with these being the two strongest correlations between *distinct *loci across the genome. When updating to Map 2, analyzing all sib pairs gave what appear to be noisier results (Fig. 1), but using only the above-mentioned four-fifths subset of sib pairs resulted in peaks more similar to those from the initial Map 1 analysis (data not shown). This suggests that this particular four-fifths subset explains in part the contrast between our Map 1 and Map 2 results, and may be more informative for linkage of *TGIF *than the full data set. Nevertheless, it seemed most appropriate to focus on results for the more comprehensive Map 2, and the full available data set.

To our knowledge, this study is the first to use ANNs for linkage analysis of quantitative traits, and the first attempt to analyze ANN weights for evidence of interactions explicitly. Our approach was able to detect linkage signals indicated by classical methods. However, optimizing network and training parameters and obtaining stable results remains challenging, especially when dealing with "noisy" real data sets. Despite these difficulties, we observed that when a trained ANN recapitulated signals from traditional analysis, correlation analysis of the weights appeared to provide some insight into locus × locus interactions. Future analysis with simulation data will be useful to systematically evaluate these methods.

## Competing interests

The author(s) declare that they have no competing interests.
